# The identification of immune genes in the milk transcriptome of the Tasmanian devil (*Sarcophilus harrisii*)

**DOI:** 10.7717/peerj.1569

**Published:** 2016-01-12

**Authors:** Rehana V. Hewavisenti, Katrina M. Morris, Denis O’Meally, Yuanyuan Cheng, Anthony T. Papenfuss, Katherine Belov

**Affiliations:** 1 Faculty of Veterinary Science, University of Sydney, Sydney, Australia; 2 Bioinformatics Division, The Walter and Eliza Hall Institute for Medical Research, Parkville, Australia; 3 Bioinformatics and Cancer Genomics, Peter MacCallum Cancer Centre, East Melbourne, Victoria, Australia

**Keywords:** Tasmanian devil, Milk, Lactation, Devil facial tumour disease, Marsupial, Immunity

## Abstract

Tasmanian devil (*Sarcophilus harrisii*) pouch young, like other marsupials, are born underdeveloped and immunologically naïve, and are unable to mount an adaptive immune response. The mother’s milk provides nutrients for growth and development as well as providing passive immunity. To better understand immune response in this endangered species, we set out to characterise the genes involved in passive immunity by sequencing and annotating the transcriptome of a devil milk sample collected during mid-lactation. At mid-lactation we expect the young to have heightened immune responses, as they have emerged from the pouch, encountering new pathogens. A total of 233,660 transcripts were identified, including approximately 17,827 unique protein-coding genes and 846 immune genes. The most highly expressed transcripts were dominated by milk protein genes such as those encoding early lactation protein, late lactation proteins, *α*-lactalbumin, *α*-casein and *β*-casein. There were numerous highly expressed immune genes including lysozyme, whey acidic protein, ferritin and major histocompatibility complex I and II. Genes encoding immunoglobulins, antimicrobial peptides, chemokines and immune cell receptors were also identified. The array of immune genes identified in this study reflects the importance of the milk in providing immune protection to Tasmanian devil young and provides the first insight into Tasmanian devil milk.

## Introduction

The Tasmanian devil (*Sarcophilus harrisii*) is the largest carnivorous marsupial in the world and belongs to the Dasyuridae family (McCallum, 2008). It is endangered due to the spread of Devil Facial Tumour Disease (DFTD) (Hawkins et al., 2009). Devils, like all other marsupial species, have a short gestation followed by an extended lactation period. Marsupial young are born at an early stage in development and do not have a functional adaptive immune system (Basden, Cooper & Deane, 1997; Old & Deane, 2000; Old & Deane, 2003). Immune compounds in the milk provide critical immune protection (Adamski & Demmer, 2000; Daly et al., 2007). Devils give birth to large litters of 20–30, after a gestation of approximately 24 days (Hesterman, Jones & Schwarzenberger, 2008), with a maximum of four pouch young (PY) attaching to the four teats in the pouch for further development. PY are permanently attached to the teat until 100 days, when they start to intermittently suckle. Beginning at approximately 105 days, the PY are left in the den while the mother scavenges for food. Lactation ceases approximately seven to eight months post parturition (Guiler, 1970).

The endangered status of the Tasmanian devils necessitates urgent research into the immune response of the species. It is notable that despite an apparent susceptibility to cancer (Canfield, Hartley & Reddacliff, 1990; Griner, 1979), a current transmissible cancer epidemic (Jones et al., 2004), and a paucity of genetic diversity due to several population bottlenecks (Brueniche-Olsen et al., 2014; Jones et al., 2004; Morris, Austin & Belov, 2013), the species is remarkably resilient to disease. Devils are scavengers and are able to digest entire rotting carcasses (Owen & Pemberton, 2005) and carry large parasite loads without showing any deleterious effects (Beveridge et al., 1975; Beveridge & Spratt, 2003). Over the past seven years we have begun to characterise the immunogenome of the devil, including characterisation of major histocompatibility complex (MHC) (Cheng et al., 2012; Siddle, Sanderson & Belov, 2007), Natural Killer (NK) cell receptors (Van Der Kraan et al., 2013), Toll-like receptors (Cui, Cheng & Belov, 2015), cytokines and immunoglobulins (Morris et al., 2015). A gap remaining in our understanding of devil immunity is the role of passive immunity in protecting the devil young while they are in the pouch. Here, we identify key milk proteins, with a focus on key immune genes, in the milk transcriptome of the Tasmanian devil, at four months of lactation. At this time, the mother is in late mid-lactation and the devil young are beginning to be left in the den, while the mother scavenges for food. During this time, PY are exposed to a range of novel pathogens within the den and in the solid food they are beginning to eat. In other marsupials it has been demonstrated that late in mid-lactation, immune compounds are upregulated to provide young with additional immune protection as they encounter novel pathogens (Adamski & Demmer, 2000; Daly et al., 2007), thus we expect the devil milk at this time to be enriched with immune compounds.

Previous studies into milk protein composition in marsupials have mostly examined single proteins or small groups of proteins (Adamski & Demmer, 2000; Daly et al., 2007; Joss et al., 2007). A single study in the tammar wallaby (*Macropus eugenii*) has looked at the protein composition of milk on a wider scale through transcriptomics (Lefevre et al., 2007). This study identified key immune genes including cathelicidins, IgA, major histocompatibility complex class II (MHC II), Ig*κ* light chain and butyrophilin in wallaby milk. Here we describe the immune gene composition of Tasmanian devil milk at mid-lactation and comment on the relevance of these findings to Tasmanian devil biology and immunity.

## Materials & Methods

### Ethics statement

The milk collection in this study was approved by The University of Sydney Animal Ethics Committee *(Animal Ethics no. 6039*).

### Sampling

Approximately 10 mL of milk was collected from a Tasmanian devil at approximately four months (∾120 days) post-parturition. The animal was held at the Australian Reptile Park, Somersby, NSW, Australia.

### RNA isolation and sequencing

The milk sample was kept on ice during transport and RNA extraction was carried out within 2–3 h after collection. Approximately 7 ml of milk was centrifuged at 2,000 × g at 4 °C for 10 min. The top fat layer was removed and the bottom layer was washed once using 10 ml PBS (pH 7.2, with 0.5 mM EDTA) followed by centrifugation at 2,000 × g and 4 °C for 10 min. The cell pellet (which would have contained neutrophils, macrophages and lymphocytes, with a lower abundance of epithelial cells and granulocytes (Young et al., 1997; Young & Deane, 2001) was recovered and RNA was extracted using 1 ml TRIzol^®^ Reagent (Life Technologies, Carlsbad, CA, USA), following the RNA extraction protocol of the manufacturer, including removal of fat, proteins and other material, and an RNase-Free DNase Set (Qiagen, Hilden, Germany) was used to remove DNA contamination from the extracted RNA. A second round of purification was performed using an RNeasy Mini Kit (Qiagen, Hilden, Germany) and quality-checked on a Bioanalyser (Agilent Technologies, Santa Clara, CA USA). The final yield of total RNA was approximately 320 ng and RNA integrity number was 7.3. Library construction and sequencing were performed by The Ramaciotti Centre (UNSW) with TruSeq chemistry on a HiSeq2000 (Illumina, San Diego, CA, USA). Approximately 22.5 million paired 100 bp reads were obtained, totalling over 44.9 Gbp of data. Reads were submitted to the NCBI Sequence Read Archive under the BioProject accession PRJNA274196and BioSample accession SUB812082. The assembled transcriptome was submitted to the Transcriptome Shotgun Assembly Sequence Database under accession GEDN00000000.

### Transcriptome assembly and annotation

RNAseq reads were assembled with the Trinity pipeline (released 10 November 2013) (Haas et al., 2013) using the default parameters. This assembly resulted in 200,829 contigs, with an N50 contig size of 2,720 bp, a mean contig length of 1,285 bp, and a transcript sum of 258.1 Mb. Functional annotation of the devil milk transcriptome was performed using the Trinotate pipeline (released 10 November 2013 (default settings): http://trinotate.github.io). In brief, BLASTp was performed using devil milk predicted ORFs as the query and the SwissProt non-redundant database (accessed 29th July 2013) as the target and the de novo transcripts aligned against the same using BLASTx (Altschul et al., 1990). HMMER v3.1b1 and Pfam v27 databases (Finn, Clements & Eddy, 2011) were used to predict protein domains, SignalP v4.1 (Petersen et al., 2011) to predict the presence of signal peptides, RNAmmer v1.2 (Lagesen et al., 2007) to predict rDNA, and TMHMM v2.0 (Krogh et al., 2001) to predict transmembrane helices within the predicted ORFs from the milk transcriptome. These transcriptome annotations were loaded into an SQLite database, and abundance estimation was performed using the RSEM v1.2.1 (default settings) (Li & Dewey, 2011) method. GO terms linked to the SwissProt entry of the best BLAST hit were used for ontology annotation. GO functional classifications and plotting was performed by WEGO (http://wego.genomics.org.cn) (Jia et al., 2006).

### Top 200 most highly expressed transcripts

The most highly expressed transcript variants were selected based on FPKM (fragments per kilobase of exon per million fragments mapped) gene expression estimation. Twenty-four transcripts of the top 200 had no BLAST hits through the Trinotate pipeline. In order to annotate these transcripts, they were further investigated using BLAST searches against the Tasmanian devil genome on ENSEMBL (release 75) (http://www.ensembl.org/index.html), or tBLASTx to GenBank nucleotide and EST collections. Transcripts that had poor BLAST hits (*E*-value >1 × 10^−10^) to SwissProt sequences were also verified using these methods.

For sequences that could not be identified using the above methods, HMMER v3.1b1 (Finn, Clements & Eddy, 2011) and SignalP v4.1 (Petersen et al., 2011) searches were used. Rfam 12.0 (Griffiths-Jones et al., 2003) and Pfam v27 (Finn et al., 2014) were used to identify conserved RNA and protein domains respectively. Finally, genes were identified with the aid of conserved flanking genes in the tammar wallaby and gray short-tailed opossum (*Monodelphis domestica*) genomes. Genes flanking the unidentified genes were identified in the devil genome. Syntenic regions in the opossum and wallaby genomes were then searched for genes with homology to the devil sequence using FGENESH+ (Solovyey, 2007). Using this process, the top 200 highly expressed transcripts from the milk transcriptome were identified and annotated.

### Phylogenetic analysis

To investigate the evolutionary relationship between the late lactation proteins (LLP) of the devil and the various marsupial species, LLP protein sequences from the tammar wallaby (LLP-A (Genbank: AAQ15117), LLP-B (Genbank: AAL85634)), gray short-tailed opossum (*Monodelphis domestica*) (LLP-B1 (Genbank: XP˙007475421), LLPB-B2 (Ensemble: ENSMODG00000017471), LLP-B3 (Ensemble: ENSMODG00000017468), LLP-B4 (Ensemble: ENSMODG00000025759)), quokka (*Setonix brachyurus*) (Genbank: AAB33234.1) and brushtail possum (*Trichosurus vulpecula*) (Genbank: AAA93179.1) were obtained for phylogenetic tree construction. Tasmanian devil protein sequences for LLP-A, LLP-B, and a homolog (named LLP-like), predicted from the devil transcripts, were used in the phylogenetic tree construction for evolutionary analysis. As the transcript for LLP-like was only partial, to obtain the full sequence the missing exons were predicted from the devil genome. The region encoding LLP-like was identified using BLAST to the devil genome. This region and devil LLP-B were used as inputs in FGENESH+ (Solovyey, 2007), to identify the missing exons for the prediction. As LLP proteins belong to the lipocalin family, the opossum lipocalin-1 (Genbank: XP˙007475462.1) was used as an outgroup. MEGA 6 (Tamura et al., 2013) was used to analyse the phylogenetic evolutionary relationships between the marsupial LLP sequences. Protein sequences were aligned using MUSCLE (Edgar, 2004) using default settings (see File S1). Using the Model Selection tool in MEGA6 the JTT model (Jones, Taylor & Thornton, 1992) was identified as the best fit and a phylogenetic tree was constructed using the maximum likelihood method based on the JTT model with 1,000 bootstrap replicates. Additional alignments were produced for novel devil genes using the ClustalW algorithm (Thompson, Higgins & Gibson, 1994) in BioEdit (Hall, 1999).

### Identification of immune transcripts

A list of immune transcripts in the milk transcriptome was generated by searching the milk transcriptome with proteins from the Immunome Database for Marsupials and Monotremes (IDMM) (Wong, Papenfuss & Belov, 2011) using tBLASTn. IDMM is a database of immune genes obtained from a number of marsupials including the tammar wallaby, gray-short tail opossum, brushtail possum, northern brown bandicoot (*Isoodon macrourus*), and the monotremes platypus *(Ornithorhynchus anatinus*) and echidna (*Tachyglossus aculeatus*). Additionally, the milk transcriptome was searched with a range of devil specific immune genes identified from the devil genome using BLAST. This included devil cytokines, immunoglobulin constant regions (Morris et al., 2015), NK cell receptors (Van Der Kraan et al., 2013), defensins (EA Jones, 2015, unpublished data) and cathelicidins (E Peel, 2015, unpublished data). The most highly expressed transcripts were selected based on FPKM expression estimates. Transcripts that had poor BLAST hits (*E*-value >1 × 10^−10^) to marsupial sequences in IDMM were verified using tBLASTx to the GenBank nucleotide collection or the devil genome on ENSEMBL.

## Results

### Transcriptome overview

A transcriptome was constructed and annotated from the total milk cells of a Tasmanian devil milk sample obtained at the end of mid-lactation. We note that Tasmanian devils are an endangered species and access to milk samples is opportunistic. In this case, a female devil was given veterinary treatment due to an injury and milk could be collected while she was anaesthetised. The total number of transcripts, including transcript variants, that were expressed in the milk transcriptome was 233,660. Excluding transcript variants, the number of transcripts was 101,399, and of these, 17,827 sequences had BLAST hits to the SwissProt non-redundant database. This number provides an estimate of the number of protein-coding genes within the Tasmanian devil milk transcriptome. Transcripts that were not protein-coding genes included non-coding RNAs, transposons, and retroelements. The transcriptome included 845 immune genes representing 4.7% of the protein-coding genes and accounted for 6.6% of the transcript expression in the transcriptome.

**Figure 1:**
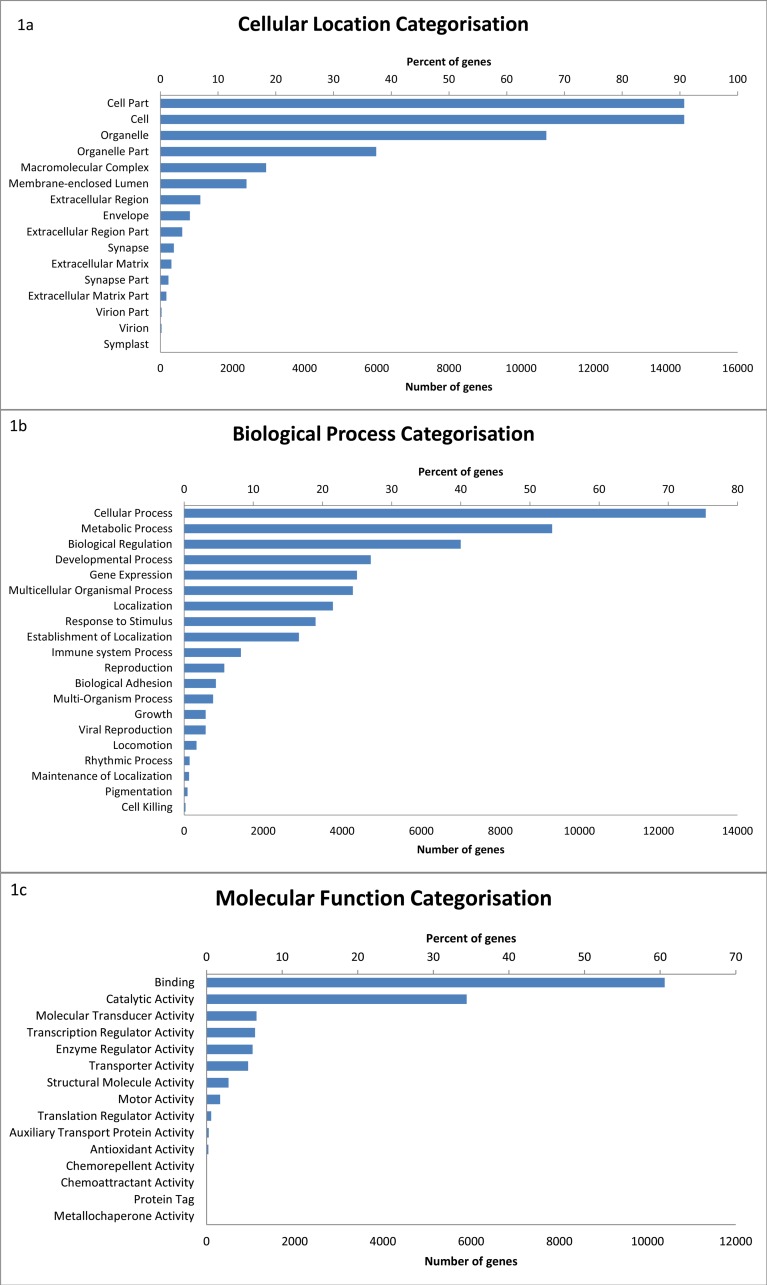
The distribution of GO terms in the devil milk transcriptome within the three level 2 GO categories of cellular location (A), biological process (B) and molecular function (C). Percentage of genes show the proportion of transcripts under each category as a percentage of all GO annotated transcripts.

Of the 17,827 sequences with BLAST hits, GO terms were assigned to 16,437 transcripts. A total of 51 level 2 GO terms were assigned (Fig. 1) (see Table S2). Within the molecular function category, binding (60.6%) and catalytic activity (34.4%) were the most common functions. Of the biological processes, genes categorised as being involved in cellular processes (75.4%) were the most common, followed by metabolic processes (53.2%), biological regulation (40.0%), and developmental processes (27.0%).

**Figure 2:**
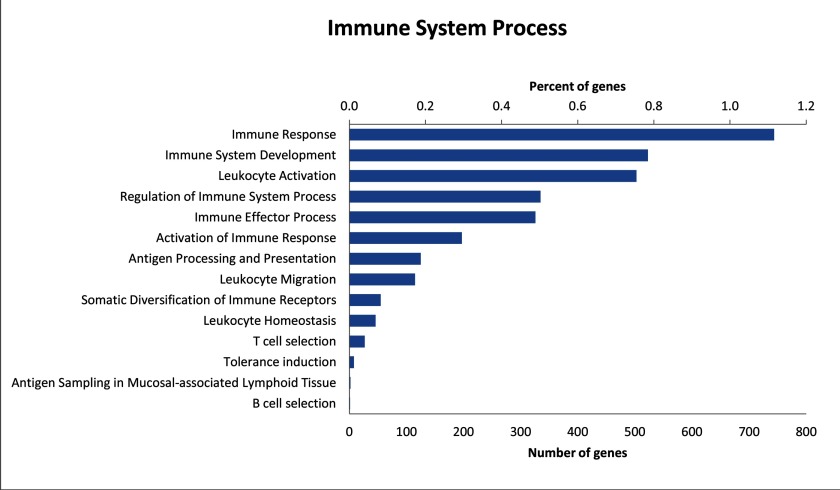
Distribution of level 3 GO terms within the level 2 category of immune system process. Percentage of genes show the proportion of transcripts under each category as a percentage of all GO annotated transcripts.

A large number of transcripts are also classified as having a role in immune system processes (8.2% or 1,341 transcripts). This number is slightly larger than the number of immune genes identified by BLAST to the IDMM database; this is likely due to a broader range of genes being classified as having a role in immune system processes within the GO annotation. Within the transcripts in the immune system process category, 14 sub-categories were represented (Fig. 2) (see Table S3). Of these transcripts, immune response genes were most common (55.5%), followed by immune system development (39.0%), leukocyte activation (37.5%) and immune effector process (24.9%).

### Most highly expressed transcripts

**Figure 3:**
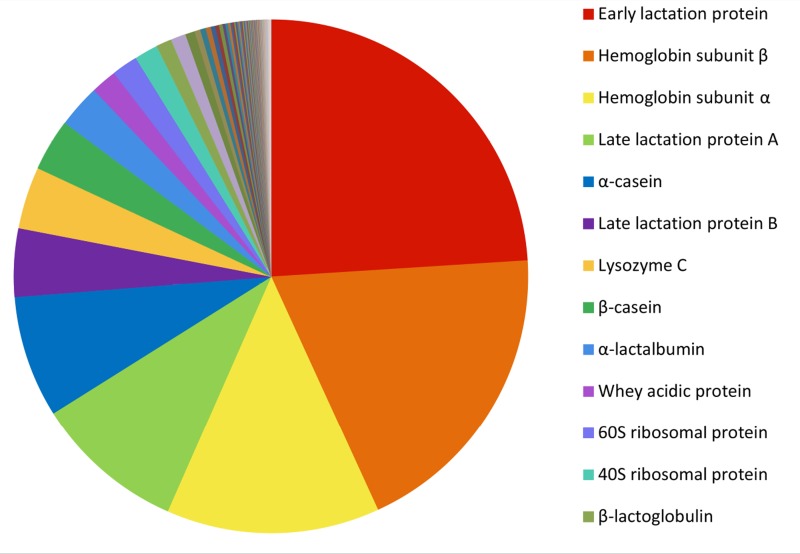
Relative abundance of the 200 most highly expressed transcripts. The FPKM gene expression for each transcript was calculated as a percentage of the total FPKM expression of the top 200 most highly expressed transcript.

The 200 most highly expressed transcripts in the Tasmanian devil milk transcriptome encode a range of nutritional milk proteins and immune proteins (Fig. 3) (see Table S4). A large proportion of most highly expressed transcripts were milk protein transcripts common across most mammals, including *α*-, *β*-, and *κ*-caseins, *α*-lactalbumin, *β*-lactoglobulin and whey acidic protein (WAP), as well as the marsupial-specific milk proteins, early lactation protein (ELP) and late lactation proteins (LLP-A and LLP-B). These together accounted for 50.21% of the total gene expression and made up the majority of the ten most highly expressed transcripts (Table 1). These proteins are mostly involved in nutrition, providing amino acids and minerals to the young, although some also have potential immune roles (Oftedal, 2012). Transcripts for proteins associated with energy metabolism, such as nicotinamide adenine dinucleotide (NADH) dehydrogenase, adenosine triphosphate synthase lipid-binding protein, and cytochrome oxidases, are present in the top 200 highly expressed transcripts.

**Table 1:** The 10 most highly expressed transcripts in the milk transcriptome.

	Transcript Id	Gene	Gene name	Relative percentage^a^	Function
1	comp129478	ELP	Early lactation protein	21.93	Nutrition: nutrient reservoir activity
2	comp127295	HBB	Haemoglobin subunit *β*	17.53	Cellular: oxygen transportation and iron binding
3	comp129479	HBA	Haemoglobin subunit *α*	12.26	Cellular: oxygen transportation and iron binding
4	comp129480	LLP-A	Late lactation protein A	8.66	Nutrition
5	comp62785	CASA1	*α*-casein	7.01	Nutrition
6	comp127104	LLP-B	Late lactation protein B	3.91	Nutrition
7	comp62784	LYZ	Lysozyme C	3.58	Immune: bacterial defence, cytolysis
8	comp129483	CSN2	*β*-Casein	2.98	Nutrition: calcium iron binding
9	comp129482	LALBA	*α*-Lactalbumin	2.49	Nutrition: lactose synthase activity/calcium iron binding
10	comp129481	WAP	Whey acidic protein	1.50	Nutrition

**Notes.**

a Relative percentage: the gene expression as a percentage of the total gene expression of the entire transcriptome.

**Figure 4:**
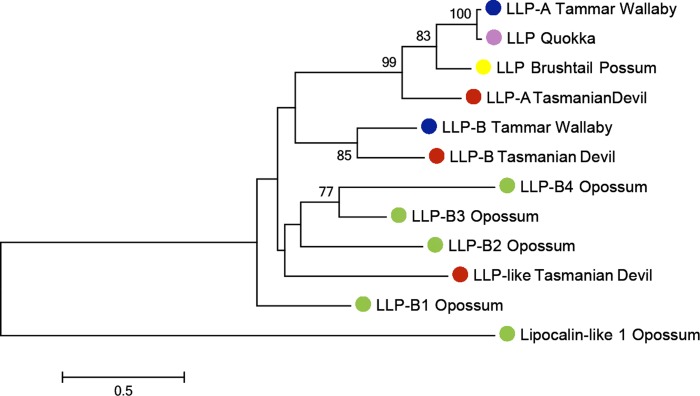
Phylogenetic tree illustrating the evolutionary relationship between LLP homologs amongmarsupials. The Tasmanian devil has three LLP homologs (LLP-A, LLP-B and LLP-like). The tree was constructed using the maximum likelihood approach and the JTT model with bootstrap support values from 1,000 bootstrap tests. Red circle, Tasmanian devil; Blue circle, Tammar wallaby; Pink circle, quokka; Green circle, gray short-tailed opossum; Yellow circle, Brushtail possum.

The highest expressed transcript was ELP, accounting for 21.93% of all transcripts. LLP-A and LLP-B were also highly expressed, as the fourth and sixth most highly expressed transcripts respectively, representing 8.66% and 3.91% of the total gene expression respectively (Table 1). The transcriptome contained a third transcript with homology to LLP-A and -B, named LLP-like, which may represent a novel Tasmanian devil LLP. To investigate the relationships between LLP-like and other Tasmanian devil and marsupial LLPs, a phylogenetic tree was constructed (Fig. 4). The number of LLP genes appears to differ between species; while a single LLP sequence has been identified in quokka and brushtail possum, two have been identified in wallaby. Through BLAST searches to the opossum genome, four opossum LLP homologs were identified in the opossum. The evolutionary relationship between Tasmanian devil LLP-like with other marsupial LLP sequences could not be definitively resolved due to weak bootstrap support. However, LLP-like does group with the other marsupial LLP sequences, and shares substantial amino acid sequence identity with other marsupial LLP sequences (23.2–44.6%), thus this sequence is likely to represent an additional LLP locus in the devil.

### Novel transcripts

**Figure 5:**
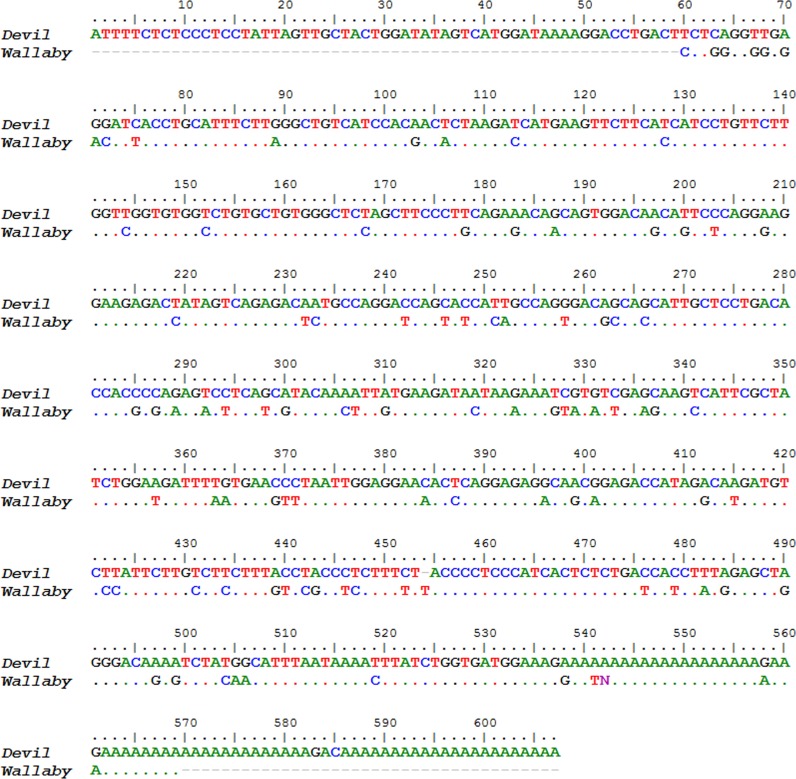
Nucleotide sequence alignment of devil Novel Gene 1 against the tammar mammary gland homolog. Dots indicate identity to devil sequence.

**Figure 6:**
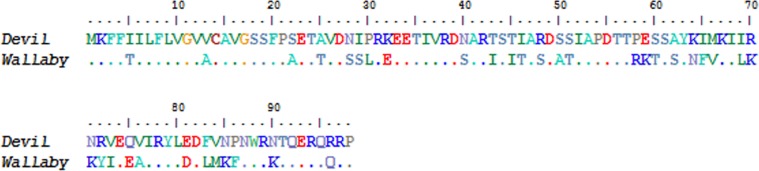
Amino acid sequence alignment of devil Novel Gene 1 against the tammar mammary gland homolog. Dots indicate identity to devil sequence.

Two novel gene transcripts were identified in the top 200, ranking 27th and 58th and accounting for 0.26% and 0.02% of the total gene expression respectively (see Table S5). Conserved motifs could not be identified in either gene. The first novel gene, which we designate here as *novel gene 1*, contained three exons that aligned to the Tasmanian devil genome, however there was no gene prediction made by the ENSEMBL annotation in this region. It did contain a predicted signal peptide cleavage site and a polyA tail, suggesting that it encodes a protein. A putative ortholog to *novel gene 1* was identified in the tammar wallaby mammary gland transcriptome (Genbank: EX196900.1). The nucleotide and protein alignments of the devil and wallaby sequences are shown in Figs. 5 and 6. The two sequences have 64% and 68% nucleotide and amino acid identity respectively. Given that a homolog could not be identified in eutherians or non-mammals and that transcripts of this gene could only be identified in milk or mammary transcriptomes, we propose that *novel gene 1* may have a marsupial-specific role in lactation.

**Figure 7:**
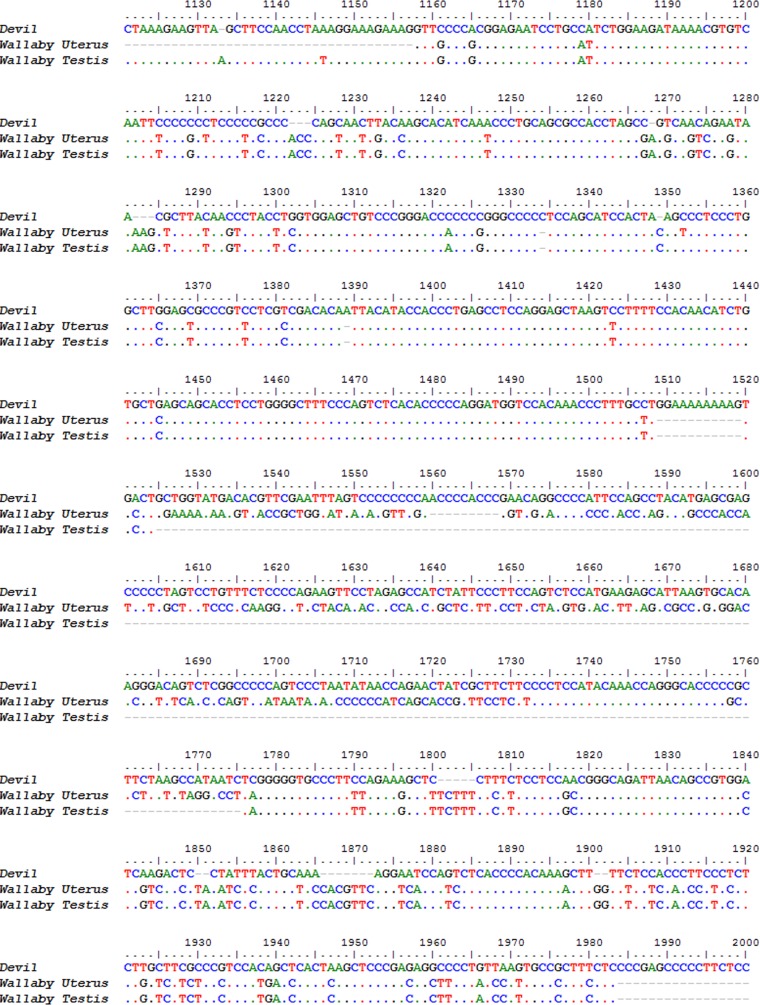
Nucleotide alignment of devil Novel Gene 2 alignment to the tammar wallaby testis and uterus homologs. Alignment begins from position 1,040 in the devil sequence. Dots indicate identity to devil sequence.

The second novel gene did not contain a signal peptide cleavage site. There were no gene predictions in the region encoding this sequence in the devil genome, nor homologs identified through HMMER searches. Additionally, the transcript is 2,072 base pairs long but does not contain any open reading frames greater than 90 residues, thus it seems unlikely to be protein-coding. Based on the length and the lack of open reading frames we speculate that it may be a long regulatory RNA. Two possible homologous sequences were identified in the tammar testis (*E* value: 4 × 10^161^) and tammar uterus (*E*-value: 0.00) transcriptomes through BLAST. Their alignment against the Tasmanian devil nucleotide sequence is shown in Fig. 7. The devil sequence has a sequence identity of 28.9% to the tammar testis and 27.8% to the tammar uterus sequences. Although this is quite low overall, there are regions within the sequences with very high identity, for example bases 1,156 to 1,499 in the devil sequence have 86.8 and 87.4% identity to the wallaby uterus and testis sequences respectively.

### Immune transcripts in the milk

**Figure 8:**
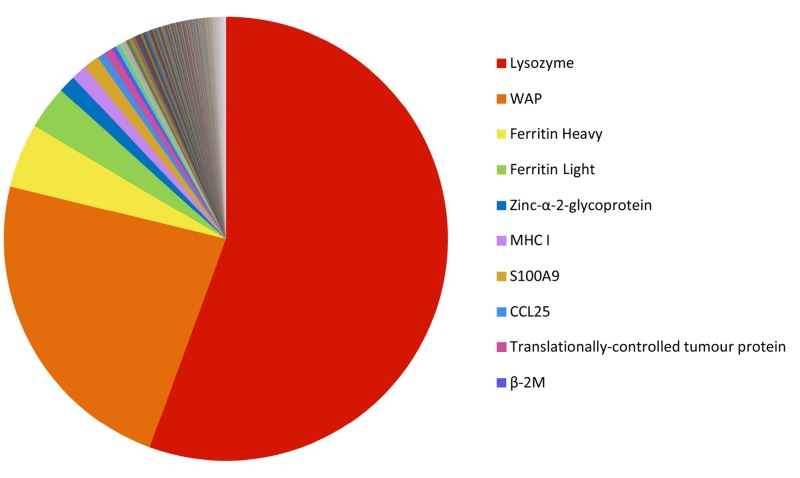
Relative abundance of the 200 most highly expressed immune transcripts. The FPKM gene expression for each immune transcript was calculated as a percentage of the total FPKM expression of the top 200 most highly expressed transcript.

There were 846 immune gene transcripts identified in the milk transcriptome, representing 6.6% of the total gene expression. The top ten are listed in Table 2, and the relative expression of the top 200 immune transcripts is shown in Fig. 8 (see Table S6). The most highly expressed immune transcripts include those encoding lysozyme C, WAP, ferritin, MHC I, S100A (calcium-binding) proteins, and CCL25.

**Table 2:** The 10 most highly expressed immune genes in the Tasmanian devil milk transcriptome.

	Transcript Id	Gene	Protein	Relative percentage^a^	Immune function
1	comp62784	LYZ	Lysozyme C	3.58	Defence again bacteria, inflammatory response. Important part of innate immunity (Nicholas et al., 1989)
2	comp129481	WAP	Whey acidic protein	1.50	Potential protease inhibitor (Nicholas et al., 1997)
3	comp62802	FTH1	Ferritin heavy subunit	0.30	Iron storage and sequestering iron (Brock, 1980; Demmer et al., 1999)
4	comp129487	FTL	Ferritin light subunit	0.21	Iron storage and sequestering iron (Brock, 1980; Demmer et al., 1999)
5	comp129504	AZGP1	Zinc-alpha-2-glycoprotein	0.08	Potential role antigen binding (Sanchez, López-Otín & Bjorkman, 1997)
6	comp129394	MHC I	Major histocompatibility complex class I	0.07	Binds to antigens and stimulation of the immune response (Chaplin, 2010)
7	comp129498	S100A9	S100 calcium binding protein A9	0.07	Important regulatory roles in inflammatory immune response. It can induce neutrophil chemotaxis and adhesion and promote phagocytosis (Ryckman et al., 2003)
8	comp62779	CLC25	Chemokine (C-C motif) ligand 25	0.06	CCL25 induces chemotaxis of thymocytes, macrophages, and dendritic cells (Vicari et al., 1997)
9	comp129492	TPT1	Translationally-controlled tumour protein	0.04	TCTP is involved in microtubule stabilization, calcium-binding activities, and apoptosis (Bommer & Thiele, 2004)
10	comp127370	B2M	*β*-2-microglobulin	0.04	*β*-chain of MHC I: serum protein associated with major MHC I heavy chain (Adamski & Demmer, 1999; Gussow et al., 1987)

**Notes.**

a Relative percentage: the gene expression as a percentage of the total gene expression of the entire transcriptome.

All four isotypes of marsupial Igs were present in the devil milk transcriptome, consistent with the gene expression profile in the tammar wallaby during mid-lactation (Lefevre et al., 2007) (Table 3). The total relative expression of all Ig transcripts was 0.01%. Transcripts encoding IgM, IgG and IgE were also present in the milk transcriptome but at lower abundance, representing 9.99 × 10^−4^%, 4.29 × 10^−4^% and 2.21 × 10^−5^% of the total expression respectively. Two Ig light chain isotypes, Ig*κ* and Ig*λ*1 were identified, with Ig*κ* being the most highly expressed (Table 3). Four Ig*λ* light chains have been identified in the Tasmanian devil (Morris et al., 2015), however only Ig*λ*1 was identified in the milk. Additional receptors involved in transfer and protection of Igs were also identified. *β*-2 microglobulin light chain (*β*-2), a receptor important for efficient transfer and uptake of IgG across the PY gut (Daly et al., 2007; Joss et al., 2009) was highly expressed in the devil milk transcriptome. Polymeric immunoglobulin receptor (pIgR), which protects Ig in the gut and has antimicrobial properties (Kaetzel, 2005), was also very highly expressed.

**Table 3:** Ig heavy and light chains expressed in the Tasmanian devil milk transcriptome.

	Transcript Id	Gene	Relative percentage^a^
1	comp128499	Igh*κ*	5.79 × 10^−3^
2	comp127776	IgA	1.96 × 10^−3^
3	comp127776	IgM	9.99 × 10^−4^
4	comp127776	IgG	4.29 × 10^−4^
5	comp12845	Ig*λ*1	1.64 × 10^−4^
6	comp78451	IgE	2.21 × 10^−5^

**Notes.**

a Relative percentage: the gene expression as a percentage of the total gene expression of the entire transcriptome.

A range of immune cell receptor genes were expressed in the milk transcriptome, providing insights into the cell types that are likely to have been in the sample at the time of collection. Phagocytes, macrophages, dendritic cells, monocytes, granulocytes, T helper and cytotoxic T cells were likely to be present based on the expression of two MHC class II *β* transcripts and MHC II *α* transcripts (Ting & Trowsdale, 2002), numerous toll-like receptors (1, 4, 5, 7, 8 and 9) (Akira, Takeda & Kaisho, 2001), *CD14*, (Taylor et al., 2005) *CD4* (Doyle & Strominger, 1987) and *CD8* (Gibbings & Befus, 2009).

Ten natural killer receptor (NKR) transcripts (Table 4) and 18 chemokine gene transcripts were identified in the milk transcriptome (Table 5). *CCL25*, *IL8*, *CXCL1L* and *CCL28* were the most highly expressed (Table 5). Antimicrobial peptides were also present (Table 6). This included four cathelicidins and three *β*-defensins.

**Table 4:** NK receptor genes identified in the Tasmanian devil milk transcriptome.

	Transcript Id	Gene	Relative percentage^a^
1	comp145309	Dig9	2.09 × 10^−3^
2	comp129464	Dig1	1.57 × 10^−3^
3	comp124809	Dig3	1.18 × 10^−3^
4	comp124376	Dig21	8.45 × 10^−4^
5	comp106701	Dig5	5.22 × 10^−4^
6	comp128578	Dig4	2.34 × 10^−4^
7	comp128741	Dig23	1.84 × 10^−4^
8	comp114623	Dig11	3.57 × 10^−5^
9	comp114366	Dig15	1.92 × 10^−5^
10	comp136274	Dig16	<1 × 10^8^

**Notes.**

a Relative percentage: the gene expression as a percentage of the total gene expression of the entire transcriptome.

**Table 5:** Chemokines expressed in the Tasmanian devil milk transcriptome.

	Transcript Id	Gene	Relative percentage^a^
1	comp62779	CCL25	0.06
2	comp124292	IL8	1.50 × 10^−2^
3	comp115841	CXCL1L	4.47 × 10^−3^
4	comp63052	CCL28	2.03 × 10^−3^
5	comp63043	CCLD13	4.32 × 10^−4^
6	comp125502	CCLD14	3.69 × 10^−4^
7	comp122269	CCL24	1.73 × 10^−4^
8	comp130761	CXCL10A	1.48 × 10^−4^
9	comp63705	CCLD4	1.05 × 10^−4^
10	comp129405	CCLD9	9.24 × 10^−5^
11	comp115375	CCLD1	8.72 × 10^−5^
12	comp121731	CCLD15	8.02 × 10^−5^
13	comp125502	CCLD6	4.97 × 10^−5^
14	comp119846	CXCL5L	4.97 × 10^−5^
15	comp60658	XCLA	4.71 × 10^−5^
16	comp132809	CXCL9	4.01 × 10^−5^
17	comp141960	CCLD2	2.53 × 10^−5^
18	comp51233	CCLD3	2.01 × 10^−5^

**Notes.**

a Relative percentage: the gene expression as a percentage of the total gene expression of the entire transcriptome.

**Table 6:** Cathelicidins and defensins expressed in the milk transcriptome.

	Transcript id	Gene	Relative percentage^a^
1	comp62677	CATH2	1.11 × 10^−3^
2	comp122064	CATH7	1.09 × 10^−4^
3	comp125957	CATH6	1.46 × 10^−4^
4	comp115402	CATH1	4.62 × 10^−5^
5	comp120278 (seq2)	*β*-defensin 1	5.58 × 10^−5^
6	comp120278 (seq4)	*β*-defensin 2	2.70 × 10^−5^
7	comp50770	*β*-defensin 5	1.57 × 10^−5^

**Notes.**

a Relative percentage: the amount of expression the gene represents as a percentage of the total gene expression the entire transcriptome.

## Discussion

In this study we have examined the milk of the Tasmanian devil for the first time. Through transcriptomic analysis of milk at mid-lactation we add to the growing body of knowledge on the composition of marsupial milk, which has previously largely focussed on just two species, wallaby and possum. We have examined the devil milk during a period of increased exposure to novel pathogens, allowing us to examine the immune components that protect young during this time.

The expression of nutritional compounds in the mid-lactation milk of the devil was very similar to what has been previously observed in marsupials. Major milk proteins, previously found to be highly expressed in eutherian and marsupial milk, including caseins, *β*-lactoglobulin, and *α*-lactalbumin, were found to be highly expressed in the devil milk, as expected. However several interesting differences were observed. Compared to the wallaby, expression of caseins, *α*-lactalbumin and *β*-lactoglobulin were considerably lower in the present study (2.49% and 0.89% respectively) than in the wallaby (4.5% and 13.4% respectively (Lefevre et al., 2007)), while the expression of ELP and LLP were far higher. ELP and LLPs are phase-specific lactation proteins unique to marsupials, being expressed in early and late lactation respectively (Demmer et al., 1998; Joss et al., 2009). In the Tasmanian devil mid-lactation milk, ELP was the most highly expressed transcript accounting for 21.93% of all transcripts while LLP-A and LLP-B represented 8.66% and 3.91% of the total gene expression respectively. The expression of these proteins was much higher in the devil milk than in the tammar wallaby mammary gland at mid-lactation, where ELP and LLP-A accounted for only 0.5% and 0.1% of transcript expression respectively, and LLP-B was not detected at mid-lactation at all (Lefevre et al., 2007). This is the first time high expression of ELP and LLP has been observed at the same time in a marsupial.

While all these proteins may play a role in nutrition (Oftedal, 2012), the roles they play in marsupial lactation has not been clearly established. It is possible that devils and wallabies are recruiting different proteins to fulfil the amino acid requirements of their PY, or that the differences in expression are due to additional functions of these proteins, for example several of these proteins are believed to play an immune role. ELP is likely to be a protease inhibitor, acting to prevent Ig degradation in the gut of the young (Pharo et al., 2012) while *β*-lactoglobulin can be digested into anti-bacterial peptides in the gut (Jenness, 1986; Pellegrini et al., 2001).

Interestingly, the transcriptome contained a third transcript with homology to *LLP-A* and-*B*, named *LLP-like*, which may represent a novel Tasmanian devil LLP. The number of LLP-related proteins discovered varied between marsupials, from one in the possum to four in the opossum. It appears this gene family is rapidly evolving and different genes may have different functions across marsupial species.

The expression of trichosurin, a marsupial-specific protein with unknown function, was much lower in the devil (0.04%) than in the wallaby (1.80%) (Lefevre et al., 2007) at mid-lactation. This finding supports the previous suggestion that the primary role of trichosurin is in enabling digestion of plant-derived phenolic compounds when marsupial young move onto a solid diet (Watson et al., 2007). As devils are omnivores, they have a much lower percentage of plant-based materials in their diet than herbivorous wallabies.

In terms of immune gene content, there were many commonalities between the Tasmanian devil milk and wallaby mammary mid-lactation transcriptomes, illustrating that similar mechanisms provide immune protection to the young at this stage. All four immunoglobulin (Ig) isotypes were identified. As in the wallaby and possum, IgA was the most highly expressed immunoglobulin in the devil mid-lactation milk, and is likely to have a key role in protecting the gut of the young (Adamski & Demmer, 2000; Daly et al., 2007). Proteins involved in transfer and protection of Igs, including WAP, B2M and pIgR were also highly expressed. Chemokines, such as CCL28, and antimicrobial peptides including cathelicidins, known to have direct antimicrobial function in milk, were identified in the devil milk. Cathelicidins in wallaby milk have been shown to be highly potent and inhibit the growth of opportunistic pathogens such as *Salmonella enterica* (Pasupuleti, Schmidtchen & Malmsten, 2012; Wang et al., 2011; Wanyonyi et al., 2013; Wanyonyi et al., 2011), thus these peptides likely play a crucial role in protecting devil young from ingested bacterial and fungal species. Macrophages, lymphocytes, and neutrophils are likely present in the milk samples at varying levels based on the presence of immune receptor molecules.

The most notable difference between the immune components of wallaby and devil mid-lactation transcriptomes was the very high expression of Lysozyme C, which plays an important role in innate immunity by breaking down glycosidic linkages in bacterial cell-wall polysaccharides, resulting in bacterial cell lysis (Nicholas et al., 1989; Piotte et al., 1997). In the devil mid-lactation milk it was the 7th most abundant transcript, while its presence was not reported in the wallaby mammary transcriptome (Lefevre et al., 2007). This may indicate that Lysozyme C is more significant in the protection of devil young than wallaby young, possibly due to the higher pathogen load in the diet of devils which is largely comprised of scavenged carcasses (Owen & Pemberton, 2005).

We note that a limitation of this study is that only one sample could be obtained (as we collected opportunistically), and thus the findings cannot be generalised across all devil milk from this period. However, previously it was shown in the tammar wallaby milk transcriptome that the composition of samples collected on the same day of lactation were highly similar (Lefevre et al., 2007). Therefore it is likely that our sample is a reasonable representation of mid-lactation milk in the devil, one of the two critical immune periods for marsupial young (Daly et al., 2007). It would also be of interest to examine the components of the first critical immune period, which is during the first 48 h after birth (Daly et al., 2007); however, this would be a highly challenging sample to obtain without risking death of the offspring and is not currently permitted in the captive animals we work with, due to the need to grow the population for release into the wild.

This study has provided valuable insight into the gene expression profile of Tasmanian devil milk during mid-lactation. We have characterised a range of immune proteins crucial for protection of devil joeys during this vulnerable stage.

## Supplemental Information

10.7717/peerj.1569/supp-1 File S1 Alignment of LLP sequences for phylogenetic tree construction

10.7717/peerj.1569/supp-2 Table S2 Allocation of genes to GO categories for cellular location, biologicalprocess and molecular function Number of genes and percentage of genes relative to all GO annotated genes within the three categories level 1 GO categories.

10.7717/peerj.1569/supp-3 Table S3 Immune gene allocation to GO “Immune Processes” categorization Number of genes and percentage of genes relative to all GO annotated genes within the immune processes categories.

10.7717/peerj.1569/supp-4 Table S4 The 200 most highly expressed transcripts in the milk transcriptome The most highly expressed transcripts including key milk protein and immune transcripts and their relative expression (FPKM).

10.7717/peerj.1569/supp-5 Table S5 Novel gene transcripts The relative expression of the two novel gene transcripts (gene expression as a percentage of the total gene expression of the entire transcriptome).

10.7717/peerj.1569/supp-6 Table S6 Immune transcripts identified in the milk transcriptome All immune transcripts identified in the milk transcriptome and their relative expression (FPKM).

## Additional Information and Declarations

### Data Availability

The following information was supplied regarding data availability. The data set supporting the results of this article is available in the short read archive repository, (SRA: SRR1793358). This Transcriptome Shotgun Assembly project has been deposited at DDBJ/EMBL/GenBank under the accession GEDN00000000. The version described in this paper is the first version, GEDN01000000.

## References

[ref-2] Adamski FM, Demmer J (1999). Two stages of increased IgA transfer during lactation in the marsupial, Trichosurus vulpecula (brushtail possum). Journal of Immunology.

[ref-1] Adamski FM, Demmer J (2000). Immunological protection of the vulnerable marsupial pouch young: two periods of immune transfer during lactation in *Trichosurus vulpecula* (brushtail possum). Developmental and Comparative Immunology.

[ref-3] Akira S, Takeda K, Kaisho T (2001). Toll-like receptors: critical proteins linking innate and acquired immunity. Nature Immunology.

[ref-4] Altschul SF, Gish W, Miller W, Myers EW, Lipman DJ (1990). Basic local alignment search tool. Journal of Molecular Biology.

[ref-5] Basden K, Cooper DW, Deane EM (1997). Development of the lymphoid tissues of the tammar wallaby *Macropus eugenii*. Reproduction Fertility and Development.

[ref-6] Beveridge I, Rickard MD, Gregory GG, Munday BL (1975). Studies on Anoplotaenia-Dasyuri Beddard, 1911 (Cestoda-Taeniidae), a parasite of Tasmanian devil—observations on egg and metacestode. International Journal for Parasitology.

[ref-7] Beveridge I, Spratt DM (2003). Parasites of carnivorous marsupials.

[ref-8] Bommer UA, Thiele BJ (2004). The translationally controlled tumour protein (TCTP). International Journal of Biochemistry & Cell Biology.

[ref-9] Brock JH (1980). Lactoferrin in human milk: its role in iron absorption and protection against enteric infection in the newborn infant. Archives of Disease in Childhood.

[ref-10] Brueniche-Olsen A, Jones ME, Austin JJ, Burridge CP, Holland BR (2014). Extensive population decline in the Tasmanian devil predates European settlement and devil facial tumour disease. Biology Letters.

[ref-11] Canfield PJ, Hartley WJ, Reddacliff GL (1990). Spontaneous proliferations in Australian marsupials—a survey and review. 1. Macropods, koalas, wombats, possums and gliders. Journal of Comparative Pathology.

[ref-12] Chaplin DD (2010). Overview of the immune response. Journal of Allergy and Clinical Immunology.

[ref-13] Cheng Y, Sanderson C, Jones M, Belov K (2012). Low MHC class II diversity in the Tasmanian devil *(Sarcophilus harrisii)*. Immunogenetics.

[ref-14] Cui J, Cheng Y, Belov K (2015). Diversity in the Toll-like receptor genes of the Tasmanian devil (Sarcophilus harrisii). Immunogenetics.

[ref-15] Daly KA, Digby M, Lefevre C, Mailer S, Thomson P, Nicholas K, Williamson P (2007). Analysis of the expression of immunoglobulins throughout lactation suggests two periods of immune transfer in the tammar wallaby (Macropus eugenii). Veterinary Immunology and Immunopathology.

[ref-16] Demmer J, Ross IK, Ginger MR, Piotte CK, Grigor MR (1998). Differential expression of milk protein genes during lactation in the common brushtail possum (Trichosurus vulpecula). Journal of Molecular Endocrinology.

[ref-17] Demmer J, Stasiuk SJ, Adamski FM, Grigor MR (1999). Cloning and expression of the transferrin and ferritin genes in a marsupial, the brushtail possum (*Trichosurus vulpecula*). Biochimica et Biophysica Acta.

[ref-18] Doyle C, Strominger JL (1987). Interaction between CD4 and Class II MHC molecules mediates cell-adhesion. Nature.

[ref-19] Edgar RC (2004). MUSCLE: multiple sequence alignment with high accuracy and high throughput. Nucleic Acids Research.

[ref-20] Finn RD, Bateman A, Clements J, Coggill P, Eberhardt RY, Eddy SR, Heger A, Hetherington K, Holm L, Mistry J, Sonnhammer ELL, Tate J, Punta M (2014). Pfam: the protein families database. Nucleic Acids Research.

[ref-21] Finn RD, Clements J, Eddy SR (2011). HMMER web server: interactive sequence similarity searching. Nucleic Acids Research.

[ref-22] Gibbings D, Befus AD (2009). CD4 and CD8: an inside-out coreceptor model for innate immune cells. Journal of Leukocyte Biology.

[ref-23] Griffiths-Jones S, Bateman A, Marshall M, Khanna A, Eddy SR (2003). Rfam: an RNA family database. Nucleic Acids Research.

[ref-24] Griner LA (1979). Neoplasms in Tasmanian devils (Sarcophilus harrisii). Journal of the National Cancer Institute.

[ref-25] Guiler ER (1970). Observations on the Tasmanian devil Sarcophilus harrisii Marsupialia dasyuridae Part 2: reproduction breeding and growth of pouch young. Australian Journal of Zoology.

[ref-26] Gussow D, Rein R, Ginjaar I, Hochstenbach F, Seemann G, Kottman A, Ploegh HL (1987). The human beta 2-microglobulin gene. Primary structure and definition of the transcriptional unit. Journal of Immunology.

[ref-27] Haas BJ, Papanicolaou A, Yassour M, Grabherr M, Blood PD, Bowden J, Couger MB, Eccles D, Li B, Lieber M, MacManes MD, Ott M, Orvis J, Pochet N, Strozzi F, Weeks N, Westerman R, William T, Dewey CN, Henschel R, Leduc RD, Friedman N, Regev A (2013). De novo transcript sequence reconstruction from RNA-seq using the Trinity platform for reference generation and analysis. Nature Protocols.

[ref-28] Hall TA (1999). BioEdit: a user-friendly biological sequence editor and analysis program for Windows 95/98/NT. Nucleic Acids Symposium Series.

[ref-29] Hawkins CE, McCallum H, Mooney N, Jones M, Holdsworth M (2009). http://www.iucnredlist.org.

[ref-30] Hesterman H, Jones SM, Schwarzenberger F (2008). Reproductive endocrinology of the largest dasyurids: characterization of ovarian cycles by plasma and fecal steroid monitoring. Part I. The Tasmanian devil (Sarcophilus harrisii). General and Comparative Endocrinology.

[ref-31] Jenness R (1986). Lactational performance of various mammalian species. Journal of Dairy Science.

[ref-32] Jia Y, Lars B, Jun W, Lin F, Hongkun Z, Yong Z, Jie C, Zengjin Z, Jing W, Shengting L, Ruiqiang L (2006). WEGO: a web tool for plotting GO annotations. Nucleic Acids Research.

[ref-33] Jones ME, Paetkau D, Geffen E, Moritz C (2004). Genetic diversity and population structure of Tasmanian devils, the largest marsupial carnivore. Molecular Ecology.

[ref-34] Jones DT, Taylor WR, Thornton JM (1992). The rapid generation of mutation data matrices from protein sequences. Computer Applications in the Biosciences.

[ref-35] Joss J, Molloy M, Hinds L, Deane E (2007). Proteomic analysis of early lactation milk of the tammar wallaby (Macropus eugenii). Comparative Biochemistry and Physiology D-Genomics & Proteomics.

[ref-36] Joss JL, Molloy MP, Hinds L, Deane E (2009). A longitudinal study of the protein components of marsupial milk from birth to weaning in the tammar wallaby (Macropus eugenii). Developmental & Comparative Immunology.

[ref-37] Kaetzel CS (2005). The polymeric immunoglobulin receptor: bridging innate and adaptive immune responses at mucosal surfaces. Immunological Reviews.

[ref-38] Krogh A, Larsson B, Von Heijne G, Sonnhammer EL (2001). Predicting transmembrane protein topology with a hidden Markov model: application to complete genomes. Journal of Molecular Biology.

[ref-39] Lagesen K, Hallin P, Rødland EA, Stærfeldt H-H, Rognes T, Ussery DW (2007). RNAmmer: consistent and rapid annotation of ribosomal RNA genes. Nucleic Acids Research.

[ref-40] Lefevre CM, Digby MR, Whitley JC, Strahm Y, Nicholas KR (2007). Lactation transcriptomics in the Australian marsupial, Macropus eugenii: transcript sequencing and quantification. BMC Genomics.

[ref-41] Li B, Dewey CN (2011). RSEM: accurate transcript quantification from RNA-Seq data with or without a reference genome. BMC Bioinformatics.

[ref-42] McCallum H (2008). Tasmanian devil facial tumour disease: lessons for conservation biology. Trends in Ecology & Evolution.

[ref-43] Morris K, Austin JJ, Belov K (2013). Low major histocompatibility complex diversity in the Tasmanian devil predates European settlement and may explain susceptibility to disease epidemics. Biology Letters.

[ref-44] Morris K, Cheng Y, Warren W, Papenfuss A, Belov K (2015). Identification and analysis of divergent immune gene families within the Tasmanian devil genome. BMC Genomics.

[ref-45] Nicholas K, Loughnan M, Messer M, Munks S, Griffiths M, Shaw D (1989). Isolation, partial sequence and asynchronous appearance during lactation of lysozyme and alpha-lactalbumin in the milk of a marsupial, the common ringtail possum (Pseudocheirus peregrinus). Comparative Biochemistry and Physiology B Biochemistry & Molecular Biology.

[ref-46] Nicholas K, Simpson K, Wilson M, Trott J, Shaw D (1997). The tammar wallaby: a model to study putative autocrine-induced changes in milk composition. Journal of Mammary Gland Biology and Neoplasia.

[ref-47] Oftedal OT (2012). The evolution of milk secretion and its ancient origins. Animal.

[ref-48] Old JM, Deane EM (2000). Development of the immune system and immunological protection in marsupial pouch young. Developmental and Comparative Immunology.

[ref-49] Old JM, Deane EM (2003). The detection of mature T- and B-cells during development of the lymphoid tissues of the tammar wallaby (Macropus eugenii). Journal of Anatomy.

[ref-50] Owen D, Pemberton D (2005). ‘Made for travelling rough’: devil ecology. Tasmanian devil: a unique and threatened animal.

[ref-51] Pasupuleti M, Schmidtchen A, Malmsten M (2012). Antimicrobial peptides: key components of the innate immune system. Critical Reviews in Biotechnology.

[ref-52] Pellegrini A, Dettling C, Thomas U, Hunziker P (2001). Isolation and characterization of four bactericidal domains in the bovine beta-lactoglobulin. Biochimica Et Biophysica Acta-General Subjects.

[ref-53] Petersen TN, Brunak S, Von Heijne G, Nielsen H (2011). SignalP 4.0: discriminating signal peptides from transmembrane regions. Nature Methods.

[ref-54] Pharo EA, De Leo AA, Renfree MB, Thomson PC, Lefevre CM, Nicholas KR (2012). The mammary gland-specific marsupial ELP and eutherian CTI share a common ancestral gene. BMC Evolutionary Biology.

[ref-55] Piotte CP, Marshall CJ, Hubbard MJ, Collect C, Grigor MR (1997). Lysozyme and alpha-lactalbumin from the milk of a marsupial, the common brush-tailed possum (Trichosurus vulpecula). Biochimica et Biophysica Acta.

[ref-56] Ryckman C, Vandal K, Rouleau P, Talbot M, Tessier PA (2003). Proinflammatory activities of S100: proteins S100A8, S100A9, and S100A8/A9 induce neutrophil chemotaxis and adhesion. Journal of Immunology.

[ref-57] Sanchez LM, López-Otín C, Bjorkman PJ (1997). Biochemical characterization and crystalization of human Zn-alpha(2)-glycoprotein, a soluble class I major histocompatibility complex homolog. Proceedings of the National Academy of Sciences of the United States of America.

[ref-58] Siddle HV, Sanderson C, Belov K (2007). Characterization of major histocompatibility complex class I and class II genes from the Tasmanian devil (Sarcophilus harrisii). Immunogenetics.

[ref-59] Solovyey VV, Balding D, Cannings C, Bishop M (2007). Statistical approaches in Eukaryotic gene prediction. Handbook of statistical genetics.

[ref-60] Tamura K, Stecher G, Peterson D, Filipski A, Kumar S (2013). MEGA6: molecular evolutionary genetics analysis version 6.0. Molecular Biology and Evolution.

[ref-61] Taylor PR, Martinez-Pomares L, Stacey M, Lin HH, Brown GD, Gordon S (2005). Macrophage receptors and immune recognition. Annual Review of Immunology.

[ref-62] Thompson JD, Higgins DG, Gibson TJ (1994). CLUSTAL W: improving the sensitivity of progressive multiple sequence alignment through sequence weighting, position-specific gap penalties and weight matrix choice. Nucleic Acids Research.

[ref-63] Ting JPY, Trowsdale J (2002). Genetic control of MHC class II expression. Cell.

[ref-64] Van Der Kraan LE, Wong ESW, Lo N, Ujvari B, Belov K (2013). Identification of natural killer cell receptor genes in the genome of the marsupial Tasmanian devil (Sarcophilus harrisii). Immunogenetics.

[ref-65] Vicari AP, Bacon KB, Zlotnik A, Figueroa DJ, Hedrick JA, Foster JS, Singh KP, Menon S, Copeland NG, Gilbert DJ, Jenkins NA (1997). TECK: A novel CC chemokine specifically expressed by thymic dendritic cells and potentially involved in T cell development. Immunity.

[ref-66] Wang J, Wong ESW, Whitley JC, Li J, Stringer JM, Short KR, Renfree MB, Belov K, Cocks BG (2011). Ancient antimicrobial peptides kill antibiotic-resistant pathogens: Australian mammals provide new options. PLoS ONE.

[ref-67] Wanyonyi S, Lefevre C, Sharp JA, Nicholas KR (2013). The extracellular matrix locally regulates asynchronous concurrent lactation in tammar wallaby (Macropus eugenii). Matrix Biology.

[ref-68] Wanyonyi S, Sharp JA, Khalil E, Lefevre C, Nicholas KR (2011). Tammar wallaby mammary cathelicidins are differentially expressed during lactation and exhibit antimicrobial and cell proliferative activity. Comparative Biochemistry and Physiology Part A: Molecular and Integrative Physiology.

[ref-69] Watson RP, Demmer J, Baker EN, Arcus VL (2007). Three-dimensional structure and ligand binding properties of trichosurin, a metatherian lipocalin from the milk whey of the common brushtail possum Trichosurus vulpecula. Biochemical Journal.

[ref-70] Wong ESW, Papenfuss AT, Belov K (2011). Immunome database for marsupials and monotremes. BMC Immunology.

[ref-71] Young L, Basden K, Cooper DW, Deane EM (1997). Cellular components of the milk of the tammar wallaby (Macropus eugenii). Australian Journal of Zoology.

[ref-72] Young LF, Deane EM (2001). Cellular composition of the late milk of the koala (Phascolarctos cinereus). Australian Journal of Zoology.

